# Freedom in bioinformatics

**DOI:** 10.3389/fgene.2014.00259

**Published:** 2014-07-31

**Authors:** Antony T. Vincent, Steve J. Charette

**Affiliations:** ^1^Institut de Biologie Intégrative et des Systèmes, Université LavalQuebec City, QC, Canada; ^2^Département de Biochimie, de Microbiologie et de Bio-informatique, Faculté des Sciences et de Génie, Université LavalQuebec City, QC, Canada; ^3^Centre de Recherche de l'Institut Universitaire de Cardiologie et de Pneumologie de QuébecQuebec City, QC, Canada

**Keywords:** freedom, open-source, proprietary tools, bioinformatics courses, software development

## What is free software?

When the young Finn Linus Torvalds and the American Richard M. Stallman respectively developed the Linux kernel and the GNU project, they probably did not anticipate the importance of their actions. The GNU/Linux operating system was created in 1992 by the fusion of the two projects. More importantly, the philosophy of freedom in the field of computing has been framed by some important rules governing the free use of software.

Many useful scientific software packages, including the European Molecular Biology Open Software Suite (EMBOSS) (Rice et al., 2000), Mothur (Schloss et al., 2009), and the Bayesian phylogenetic reconstruction tool (PhyloBayes) (Lartillot et al., 2009), are covered by GNU general public licenses (GNU GPL) that make their code freely available for everyone. GNU GPL allow for the continuous evolution of code, even if the developers are no longer involved. Science, including the field of genomics, is rapidly changing, and the tools researchers rely on must keep pace. The scientific community is best placed to know exactly what it needs in terms of bioinformatics tools. Moreover, the free software philosophy allows everyone to introduce new “flavors” for new analyses, which allows free software to evolve and adapt.

## The outbreak in proprietary bioinformatics tools

There has been an outpouring of proprietary bioinformatics software in the past few years. The term proprietary means that the code is non-free and is usually a lucrative source of income. Licenses for such software products can be very expensive. Most academic laboratories depend on grants to operate, and an increasing number of them use bioinformatics at various levels. The money required to acquire proprietary software is thus no longer available for the main purpose of research, that is, advancing scientific knowledge.

Why do proprietary software products exist, and why are they so attractive to some users? Proprietary software products, like Geneious (Biomatters Ltd., Auckland, New Zealand), CLC Genomics Workbench (CLC bio, Aarhus, Denmark) and Sequencher (Gene Codes Corporation, Ann Arbor, MI, USA), usually have attractive graphical user interfaces and combine many bioinformatics tools. Most current bioinformatics tools work in command line and, for many scientists, one of the scariest things is to work with a terminal. An important point is that no programming skills are needed with most proprietary software suites, just some basic computer abilities. Moreover, if there are any problems, companies generally provide technical support, which make users feel safe. In addition, unlike open-source applications, most of which are only available on a UNIX-based system, some proprietary software products work on all operating systems. In other words, they digest bioinformatics for everyone.

However, there are several problems with proprietary software. First, most of the bioinformatics tools in non-free graphical interfaces are, in fact, freely available. The companies that sell these proprietary products are thus making money on the back of freedom and, importantly, decrease the reference ability of the free software included in the proprietary interface by making it obligatory to refer directly to the propriety code used in studies. Moreover, it is, in most cases, impossible to control the version and to benchmark the free software packages used by these proprietary tools. Second, reproducible results are important in science. If a proprietary software product is used to analyze results, this forces other scientists to use the same proprietary software. How can we know if the results are correct when it is not possible to access the code and the algorithms? The biological sciences recently entered a new era where DNA sequencing has become increasingly available (Chain et al., 2009). Consequently, the use of bioinformatics tools is more necessary now than ever before. It is important to be able to conduct quality experiments and then have confidence in the results in order to avoid too many errors in public databases. Moreover, the purchase price and/or user fees of proprietary bioinformatics tools can have a dampening effect on research in developing countries. Open-source software, on the other hand, has the opposite effect since it is much more flexible and, more importantly, is totally free.

## How to conserve our freedom in bioinformatics

What can we do? The answer is simple. Researchers should use open-source tools instead of proprietary software products for their analyses. There are free bioinformatics tools for almost all applications. It is easy to find a list of open-source applications for biological sciences on the Internet.

Open-source application developers should, however, consider the following points when they are designing their applications: (i) look through the eyes of users and try to develop user-friendly applications, (ii) create tools that can be used on multiple platforms, and (iii) simplify the management of dependencies. However, in most cases, developing bioinformatics tools is not the main focus of laboratories, and those responsible for projects do not have the time to improve the interfaces. The increased use of bioinformatics in studies should be backed by sources of funding to actively support the development of free bioinformatics tools. Good examples of user-friendly open-source applications are Artemis (Rutherford et al., 2000) and Unipro UGENE (Okonechnikov et al., 2012), which are integrative, have an attractive graphical user interface, and are available for all operating systems.

Concretely, bioinformatics courses should embrace open-source and free applications and, above all, promote the importance of using them. Ideally, bioinformatics programs should be based on the use of open-source resources, which will necessarily enhance academic independence and freedom. Students are our future bioinformaticians and by acquainting them with open-source software they will be more likely to develop free software and introduce new standards in the field. We think bioinformatics notions should be introduced into all biological undergraduate programs, not solely bioinformatics programs. For example, here at Université Laval, microbiology and biochemistry students must take a mandatory bioinformatics course in which they are introduced, among other things, to public domain primary, secondary and specialized databases, sequence alignments, genome assembly, phylogenetic analyses, protein structure determinations, and molecular docking using open-source applications. Our undergraduate students can also continue on to a second more advanced course if they so desire.

Lastly, structured and inviting networks would be a good way of disseminating open-source bioinformatics tools and would provide important information such the newest software and publications. Improving the accessibility of free user-adapted tools will help demystify bioinformatics and, as such, contribute to spreading the philosophy of sharing and freedom.

### Conflict of interest statement

The authors declare that the research was conducted in the absence of any commercial or financial relationships that could be construed as a potential conflict of interest.
